# Left Lateral Cervical Mass with Draining Sinuses

**DOI:** 10.1155/2019/7838596

**Published:** 2019-07-25

**Authors:** Stylianos A. Michaelides, George D. Bablekos, Avgerinos-Romanos Michailidis, Efthalia Gkioxari, Stephanie Vgenopoulou, Maria Chorti

**Affiliations:** ^1^Department of Occupational Lung Diseases and Tuberculosis, “Sismanogleio- Amalia Fleming” General Hospital, 1 Sismanogleiou Str, 15126, Attiki, Maroussi, Athens, Greece; ^2^Departments of Biomedical Sciences, Nursing and Physiotherapy, University of West Attica, Campus 1, Attiki, Egaleo, Agiou Spiridonos, 12243 Athens, Greece; ^3^Second Department of Thoracic Medicine, “Sismanogleio- Amalia Fleming” General Hospital, 1 Sismanogleiou Str, 15126, Attiki, Maroussi, Athens, Greece; ^4^Department of Surgical Pathology, “Sismanogleio- Amalia Fleming” General Hospital, 1 Sismanogleiou Str, 15126, Attiki, Maroussi, Athens, Greece

## Abstract

The aim of the present study is to describe an uncommon case of tuberculous lymphadenitis (TL) in a symptomless 89-year-old male smoker patient, who presented at the emergency department of our hospital with left lateral cervical swelling with draining sinuses. No other clinical symptoms or physical findings were observed at admission. An elevated erythrocyte sedimentation rate (ESR) and a small calcified nodule in chest CT were the only abnormal findings. Pus samples from sinuses were examined and confirmed tuberculosis which was in agreement with surgical pathology of lymph nodes. A four- (4-) drug antituberculous regimen was administered. After an initial remission of his symptoms, the patient presented an exacerbation of the cervical swelling with draining sinuses necessitating addition of oral steroids. TL can be symptomless presenting a paradoxical reaction during treatment. The uniqueness of our case lies in the patient's advanced age, which is uncommon with cervical lymphadenopathy as a form of extrapulmonary tuberculosis, as well as in the administration of oral steroids to resolve the neck's clinical deterioration. The patient had a complete recovery and was free of disease after completion of his six-month antituberculous chemotherapy.

## 1. Introduction

Tuberculous lymphadenitis (TL) or scrofula was firstly described by Hippocrates [1]. During the 18^th^ century, tuberculous cervical lymphadenitis was known as the “king's evil” which was believed to be cured by the royal touch [2, 3]. TL seems to be more common in females than in males with the Asian and black women showing an increased risk for this type of infection [4]. Cervical lymphadenopathy, which is the most common form of extrapulmonary tuberculosis, is estimated to mainly appear between the ages of 20 and 40 years [5]. Other studies also report that peripheral TL is usually presented in young adults [6–8]. Particularly, for the USA and the UK, TL is frequently diagnosed between the ages of 25 and 50 years [9, 10].

We present the case of a left lateral cervical lymphadenopathy with draining sinuses, resulting from tuberculous infection. Its uniqueness lies in the complete absence of any constitutional symptom of tuberculous disease except the cervical swelling with draining sinuses, in the patient's advanced age being uncommon for this particular condition, and in the use of oral steroids when a paradoxical deterioration, after an initial remission, of the patient's neck condition during antituberculous treatment, was observed. The age and the robust response of the patient to steroid therapy, while under antituberculous treatment, despite the conflicting opinions in the literature [11], are underlined for management of similar conditions.

## 2. Case Presentation

**Figure 1:**
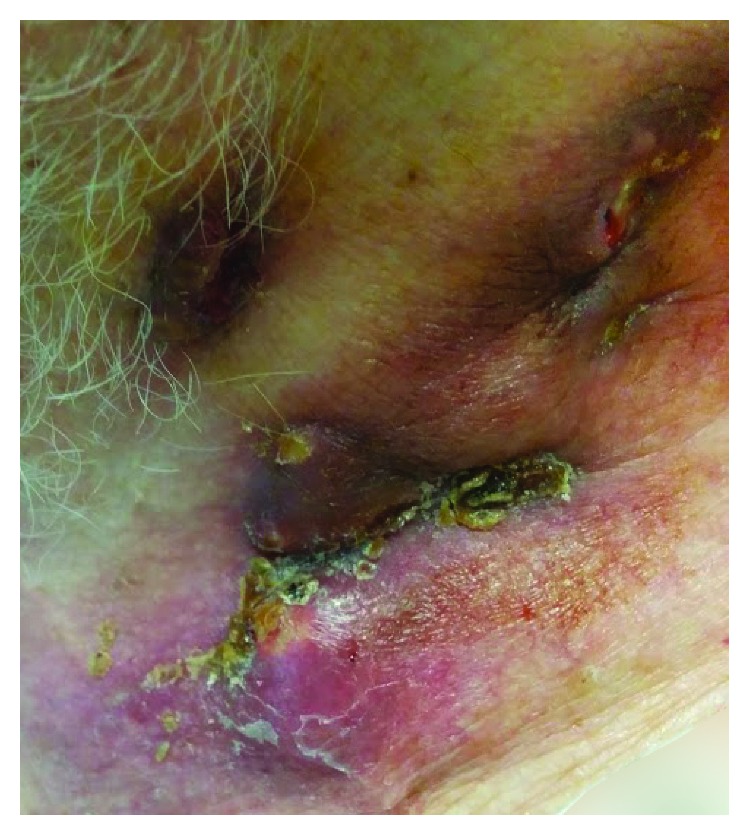
Lateral cervical area of patient with swollen and draining lymph nodes.

**Figure 2:**
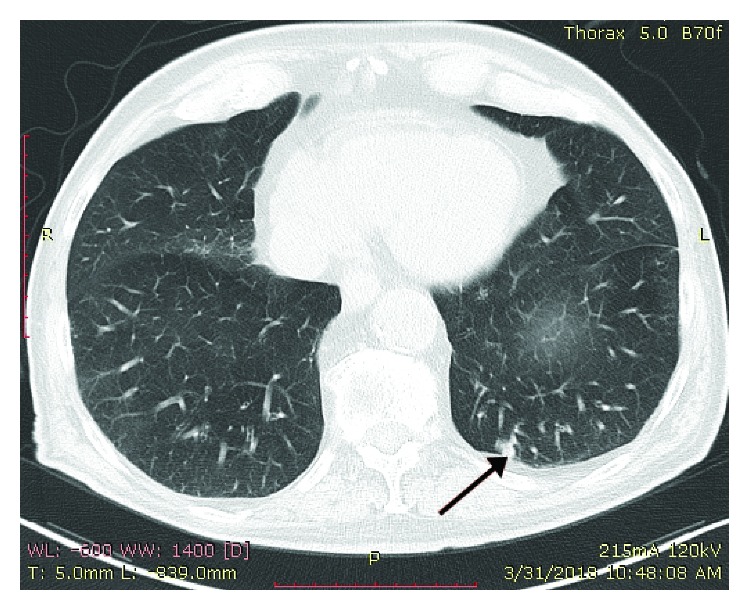
Chest CT image showing small (3 mm) nodules (black arrow) in the apical-posterior segment of the left upper lobe.

**Figure 3:**
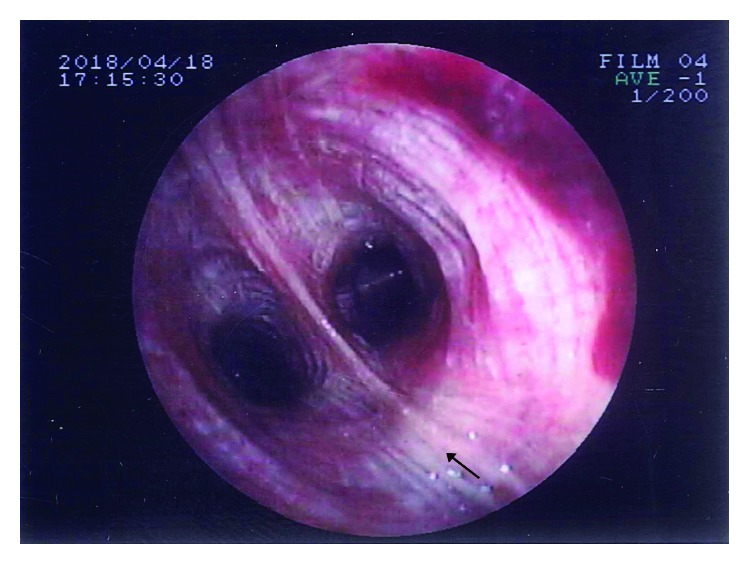
Small area with anthracotic discoloration (black arrow) in the posterior segment of the right upper lobe.

**Figure 4:**
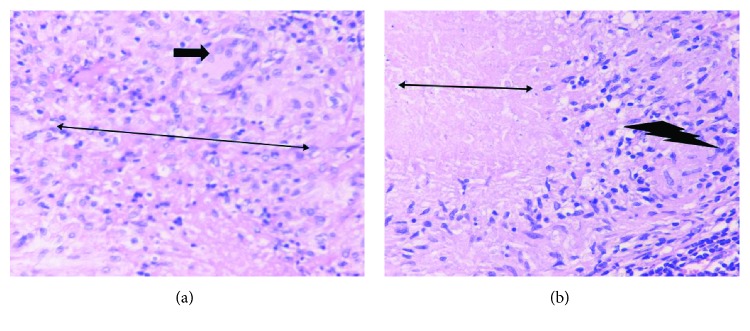
(a) Tuberculous granuloma with collections of epithelioid macrophages (thin two-direction black arrow) and Langhans giant cells (simple thick black arrow) H-EX400. (b) Central coagulative necrosis (thin two-direction black arrow) with a rim of epithelioid cells (“lightning” black arrow) H-EX100.

An 89-year-old male smoker patient presented at the emergency department of our hospital with left, deep purplish cervical swelling with draining sinuses. The patient reported that three months ago, painless erythematous nodular swellings appeared in the left lateral neck area. No respiratory symptoms, fever, or weight loss were reported except for occasional dry cough and mucous expectoration since he had been a lifelong smoker of 70 pack-years. The patient was a taxi and bus driver. His past medical history included surgery for benign prostate hypertrophy 15 years ago, as well as an unscheduled visit to the emergency department of our hospital, 6 months ago, due to an acute hypertensive episode associated with pulmonary edema, being treated by intravenous diuretics. Since then, he had been on valsartan-hydrochlorothiazide therapy (16 mg + 12.5 mg), once daily. On examination, the patient, a tall and thin man, (BMI = 19.6 Kg/m^2^), with normal skin color, had a normal physical appearance except for his left lateral neck lesion including draining tract sinuses with purulent material (Figure 1) and scarce low-pitched expiratory wheezes. The cardiovascular examination showed a mild systolic murmur in the right second intercostal space compatible with moderate aortic valve regurgitation. On admission, the blood pressure (BP) and heart rate (HR) were 145/70 mmHg and 86 beats per minute, respectively, while blood and biochemistry tests were as follows: Hct = 37.3%; Hb = 13.8 g/dL; MCV = 81.7 fL; MCH = 29.6 pg; PLT = 214 × 10^3^/*μ*L; WBC = 6.3 × 10^3^/*μ*L (leukocyte type: NEU% = 70%, LYM% = 20%, MON% = 7%, and EOS = 3%); INR = 1.22; serum glucose = 101 mg/dL; urea = 39 mg/dL; creatinine = 1.02 mg/dL; Na^+^ = 139 mmol/L; K^+^ = 4.2 mmol/L; and erythrocyte sedimentation rate (ESR) = 52 mm/1st hour. The Mantoux test showed 10 mm induration, and his chest X-ray had no significant findings. The computed tomography (CT scan) showed minimal fibrous bands in the upper zone area of the right upper lobe, while a small calcified nodule (3 mm diameter) was also found in the periphery of the apical-posterior segment of the left upper lobe (Figure 2). Fiberoptic bronchoscopy revealed a diffusely red and hyperemic bronchial mucosa, particularly intense in the segments of the right upper lobe, with the presence of some small anthracotic areas (Figure 3). Bronchoalveolar lavage fluid (BAL) obtained from the apical-posterior segment of the left upper lobe showed increased (>70%) lymphocytes, 15% neutrophils, few (<8%) macrophages, and scarcity of bronchial epithelial cells with no evidence of malignancy. Direct smear for acid-fast bacilli was also negative. The surgical biopsy and the microscopic examination of the cervical lymph node showed epithelial granuloma with central coagulative (caseous) necrosis and the presence of *M. tuberculosis* complex, respectively. In addition, the microscopic examination of the purulent content of draining sinuses in the patient's neck also revealed the presence of *M. tuberculosis* complex. Final diagnosis for TL was established by histopathologic examination (Figures 4(a) and 4(b)).

The patient was started on a four- (4-) drug antituberculous regimen. One month after the initiation of the antituberculous regimen, and after an initial remission regarding the magnitude of the patient's neck growth, he experienced an enlargement of his lymph nodes with reappearance of draining sinuses. It was then decided to add oral steroids (20 mg prednisolone daily). Four weeks after steroid administration, there was a spectacular, almost complete remission, of his neck growth. The antituberculous treatment was continued for 6 months, and the patient was free of disease after its completion.

## 3. Discussion

Extrapulmonary tuberculosis is most commonly diagnosed in peripheral lymph nodes [12]. In our case, the occasional cough and mucous expectoration were nonspecific symptoms for tuberculous disease (TB). Due to lateral neck mass appearance, the differential diagnosis may include neoplastic, infectious or immunologic diseases, sarcoidosis, abscess, tuberculosis, brucellosis, syphilis, toxoplasmosis, cat-scratch disease, and fungal infections [13].

The presence of draining sinuses with purulent material was suggestive of infectious, suppurative conditions such as pyogenic abscess [14] or other infective processes. In our patient, diagnosis of TL was based either on the identification of *Mycobacterium tuberculosis* in the pus of the draining sinuses of the lymph node or on the histopathologic examination of the lymph node showing a typical pathology of tuberculosis. For the accurate diagnosis of cervical TL, the contribution of fine-needle aspiration cytology (FNAC), while avoiding any complication associated with the open biopsy, is reported in the relevant literature [15, 16]. The use of fine-needle aspiration cytology in diagnosis of extrapulmonary tuberculous lymphadenitis was also reported by previous studies [12, 17–19], with granulomatous inflammation on fine-needle aspiration cytology to be strongly indicative for tuberculosis infection [18]. However, excisional biopsy compared to the fine-needle aspiration technique seems to be more sensitive and safer in diagnosis of peripheral TL [17], particularly when multiple lymph nodes should be examined [20]. Another issue to be discussed is the exacerbation of our patient's neck growth one month after induction of anti-TB treatment, something which is not an unusual event during therapy of TL, characterized as a paradoxical reaction (PR). Paradoxical reaction is a transitory deterioration of the clinical and/or radiological condition although the patient is under appropriate anti-TB therapy [21–23], and it is interpreted by two different proposed theories: the first lies in a delayed immune activation and the second one in a hypersensitivity reaction to the antigen released from dying mycobacteria [24]. Predictive risk factors for paradoxical reaction during treatment for cervical lymph node TB are lymph node size ≥ 3 cm and extralymph node TB (multivariate and univariate analyses), as well as sweating (univariate analysis) [25]. Age, antigenic load, lymphocyte count, inflammatory status, and vitamin D status are also independent risk factors for paradoxical reaction, when under treatment for extrapulmonary TB [22]. Moreover, baseline anemia, hypoalbuminemia, lymphopenia, and a greater change in lymphocyte count are independent risk factors for PR during therapy for pulmonary TB [26]. The incidence of paradoxical reaction, during anti-TB curative treatment, ranges from 6% to 30% [27–29] with the respiratory system, central nervous system, and lymph nodes to be mainly affected [23, 30]. In TL, paradoxical reaction usually occurs within two months, ranging from 14 to 270 days, following administration of anti-TB medication [23]. Immunotherapy with steroids or an antitumor necrosis factor-alpha (anti-TNF-a) inhibitor may help to resolve the paradoxical reaction of lymph nodes during treatment by inhibiting granuloma formation interfering with the penetration of anti-TB chemotherapy [31–33]. According to the relevant literature, when diagnosis of TL is established and adequate anti-TB regimen is administered, a relapse in adenitis or appearance of draining sinuses could be expected and managed by adding oral steroids along with the anti-TB medication [32], despite steroids not being prescribed for TL treatment [34], except for local discomfort [20]. The use of corticosteroids while receiving therapy for TL seems to resolve paradoxical reaction and improves the clinical outcome [30, 35–39]. Although steroids seem to be particularly effective against paradoxical reaction during appropriate treatment for intracranial tuberculomas [21, 32], TB meningitis [40], and pleural TB [41], another study showed no significant clinical response [42]. The rationale for the use of corticosteroids is based on the reduction of the inflammation process [30]. Specifically, for neuro-TB, the administration of corticosteroids reduces cerebral edema by also influencing cerebral vasculature [43], while for peripheral lymph node TB, the benefit of corticosteroid therapy seems to be less clear [44]. Nonetheless, a more recent comparative study showed that the routine addition of corticosteroids for the first 3-4 months in tapering dosage, even without appearance of paradoxical reaction, in patients receiving standard medication for cervical lymph node tuberculosis, is safe with better clinical outcome and complete relief of symptoms [38].

Also, surgical therapy is the method of choice to manage cervical TL attributed in nontuberculous mycobacteria, specifically in children, due to better response compared to three months of two-drug antituberculous chemotherapy [45–47].

The learning points, useful in clinical practice, from our case, can be presented as follows: (i) the patient belonged to an age group where cervical TL is particularly uncommon as a form of extrapulmonary tuberculosis, (ii) the exacerbation of the patient's neck lymphadenitis was resolved by oral steroids, and (iii) the patient was in complete recovery and free of disease after accomplishment of his 6 month antituberculous chemotherapy.

In conclusion, TL should always be among the differential diagnoses of lymph node enlargement even when no constitutional symptoms exist, as in the presented case. When diagnosis is established and the indicated antituberculous treatment is administered, a relapse in adenitis or draining sinuses presentation is not considered as treatment failure but this effect could be expected and can be managed by adding oral steroids along with the antituberculous medication.

## References

[1] Grzybowski S., Allen E. A. (1995). History and importance of scrofula. *The Lancet*.

[2] Moulis G., Martin-Blondel G. (2012). Scrofula, the king’s evil. *Canadian Medical Association Journal*.

[3] Murray J. F., Rieder H. L., Finley-Croswhite A. (2016). The king’s evil and the royal touch: the medical history of scrofula. *International Journal of Tuberculosis and Lung Disease*.

[4] Polesky A., Grove W., Bhatia G. (2005). Peripheral tuberculous lymphadenitis: epidemiology, diagnosis, treatment and outcome. *Medicine*.

[5] Golden M. P., Vikram H. R. (2005). Extrapulmonary tuberculosis: an overview. *AmFam Physician*.

[6] Enarson D. A., Ashley M. J., Grzybowski S., Ostapkowicz E., Dorken E. (1980). Non-respiratory tuberculosis in Canada. Epidemiologic and bacteriologic features. *American Journal of Epidemiology*.

[7] Farer L. S., Lowell A. M., Meador M. P. (1979). Extrapulmonary tuberculosis in the United States. *American Journal of Epidemiology*.

[8] Rieder H. L., Snider D. E., Cauthen G. M. (1990). Extrapulmonary tuberculosis in the United States. *American Review of Respiratory Disease*.

[9] Alvarez S., McCabe W. R. (1984). Extrapulmonary tuberculosis revisited: a review of experience at Boston city and other hospitals. *Medicine*.

[10] Monie R. D., Hunter A. M., Rocchiccioli K. M., White J. P., Campbell I. A., Kilpatrick G. S. (1982). Management of extra-pulmonary tuberculosis (excluding miliary and meningeal) in south and west Wales (1976-8). *BMJ*.

[11] Fontanilla J.-M., Barnes A., von Reyn C. F. (2011). Current diagnosis and management of peripheral tuberculous lymphadenitis. *Clinical Infectious Diseases*.

[12] Jha B. C., Dass A., Nagarkar N. M., Gupta R., Singhal S. (2001). Cervical tuberculous lymphadenopathy: changing clinical pattern and concepts in management. *Postgraduate Medical Journal*.

[13] Asano S. (2012). Granulomatous lymphadenitis. *Journal of Clinical and Experimental Hematopathology*.

[14] Horváth T., Horváth B., Varga Z. (2015). Severe neck infections that require wide external drainage: clinical analysis of 17 consecutive cases. *European Archives of Oto-Rhino-Laryngology*.

[15] Moualed D., Robinson M., Qureishi A., Gurr P. (2018). Cervical tuberculous lymphadenitis: diagnosis and demographics, a five-year case series in the UK. *Annals of The Royal College of Surgeons of England*.

[16] Rammeh S., Romdhane E., Arfaoui Toumi A. (2018). Efficacy of fine-needle aspiration cytology in the diagnosis of tuberculous cervical lymphadenitis. *Acta Cytologica*.

[17] Artenstein A. W., Kim J. H., Williams W. J., Chung R. C. Y. (1995). Isolated peripheral tuberculous lymphadenitis in adults: current clinical and diagnostic issues. *Clinical Infectious Diseases*.

[18] Lau S. K., Wei W. I., Hsu C., Engzell U. C. G. (1990). Efficacy of fine needle aspiration cytology in the diagnosis of tuberculous cervical lymphadenopathy. *Journal of Laryngology & Otology*.

[19] Lau S.-K., Wei W. I., Kwan S., Yew W.-W. (1991). Combined use of fine-needle aspiration cytologic examination and tuberculin skin test in the diagnosis of cervical tuberculous lymphadenitis: a prospective study. *Archives of Otolaryngology—Head and Neck Surgery*.

[20] Blaikley J. F., Khalid S., Ormerod L. P. (2011). Management of peripheral lymph node tuberculosis in routine practice: an unselected 10-year cohort. *International Journal of Tuberculosis and Lung Disease*.

[21] Rakotoson J. L., Rakotomizao J. R., Andrianasolo R. L., Rakotoharivelo H., Andrianarisoa A. C. F. (2011). Adénopathie paradoxale au cours du traitement d’une tuberculose cavitaire chez un malade immunocompétent. *Revue de Pneumologie Clinique*.

[22] Barr D. A., Coussens A. K., Irvine S. (2017). Paradoxical upgrading reaction in extra-pulmonary tuberculosis: association with vitamin D therapy. *International Journal of Tuberculosis and Lung Disease*.

[23] Cheng V., Ho P., Lee R. (2002). Clinical spectrum of paradoxical deterioration during antituberculosis therapy in non-HIV-infected patients. *European Journal of Clinical Microbiology & Infectious Diseases*.

[24] Smaoui S., Mezghanni M. A., Hammami B. (2015). Tuberculosis lymphadenitis in a southeastern region in Tunisia: epidemiology, clinical features, diagnosis and treatment. *International Journal of Mycobacteriology*.

[25] Chahed H., Hachicha H., Berriche A. (2017). Paradoxical reaction associated with cervical lymph node tuberculosis: predictive factors and therapeutic management. *International Journal of Infectious Diseases*.

[26] Cheng S. L., Wang H. C., Yang P. C. (2007). Paradoxical response during anti-tuberculosis treatment in HIV-negative patients with pulmonary tuberculosis. *International Journal of Tuberculosis and Lung Disease*.

[27] Campell I. A., Dyson A. J. (1977). Lymph node tuberculosis: a comparison of various methods of treatment. *Tubercle*.

[28] Al-Majed S. A. (1996). Study of paradoxical response to chemotherapy in tuberculous pleural effusion. *Respiratory Medicine*.

[29] Memish Z. A., Mah M. W., Al Mahmood S., Bannatyne R. M., Khan M. Y. (2000). Clinico-diagnostic experience with tuberculous lymphadenitis in Saudi Arabia. *Clinical Microbiology and Infection*.

[30] Thorve S., Patil N., Mandilwar S., Vora A. (2017). Loss of vision and hearing in a case of cervical lymph node tuberculosis: a rare paradoxical reaction. *Lung India*.

[31] Park K.-H., Cho O.-H., Chong Y. P. (2010). Post-therapy paradoxical response in immunocompetent patients with lymph node tuberculosis. *Journal of Infection*.

[32] Hawkey C. R., Yap T., Pereira J. (2005). Characterization and management of paradoxical upgrading reactions in HIV-uninfected patients with lymph node tuberculosis. *Clinical Infectious Diseases*.

[33] Blackmore T. K., Manning L., Taylor W. J., Wallis R. S. (2008). Therapeutic use of infliximab in tuberculosis to control severe paradoxical reaction of the brain and lymph nodes. *Clinical Infectious Diseases*.

[34] Centers for Disease Control (2003). Treatment of tuberculosis.

[35] Bhattacharya A., Mukherjee S. (2017). Paradoxical reaction in the form of pleural effusion after onset of anti-tuberculous medication for tubercular lymphadenitis. *Clinical Medicine*.

[36] Singh A., Rahman H., Kumar V., Anila F. (2013). An unusual case of paradoxical enlargement of lymph nodes during treatment of tuberculous lymphadenitis in immunocompetent patient and literature review. *American Journal of Case Reports*.

[37] Cruz A. T., Hernandez J. A. (2016). Tuberculosis cervical adenitis: management dilemmas. *Pediatric Infectious Disease Journal*.

[38] Bunkar M. L., Agnihotri S. P., Gupta P. R., Arya S. (2016). Add-on prednisolone in the management of cervical lymph node tuberculosis. *Indian Journal of Tuberculosis*.

[39] Shah I., Chilkar S., Patil M., Ali U. (2012). Acute respiratory distress during paradoxical reaction to antituberculous therapy in an 8-month-old child. *Lung India*.

[40] Schoeman J. F., Van Zyl L. E., Laubscher J. A., Donald P. R. (1997). Effect of corticosteroids on intracranial pressure, computed tomographic findings, and clinical outcome in young children with tuberculous meningitis. *Pediatrics*.

[41] Jung J. W., Shin J. W., Kim J. Y. (2011). Risk factors for development of paradoxical response during anti-tuberculosis treatment in HIV-negative patients with pleural tuberculosis. *Tohoku Journal of Experimental Medicine*.

[42] Geri G., Passeron A., Heym B. (2013). Paradoxical reactions during treatment of tuberculosis with extrapulmonary manifestations in HIV-negative patients. *Infection*.

[43] Kalita J., Misra U. K. (2001). Effect of methyl prednisolone on sensory motor functions in tuberculous meningitis. *Neurology India*.

[44] Dooley D. P., Carpenter J. L., Rademacher S. (1997). Adjunctive corticosteroid therapy for tuberculosis: a critical reappraisal of the literature. *Clinical Infectious Diseases*.

[45] Lindeboom J. A., Kuijper E. J., Bruijnesteijn van Coppenraet E. S., Lindeboom R., Prins J. M. (2007). Surgical excision versus antibiotic treatment for nontuberculous mycobacterial cervicofacial lymphadenitis in children: a multicenter, randomized, controlled trial. *Clinical Infectious Diseases*.

[46] Zaharia A., Eidlitz-Markus T., Haimi-Cohen Y., Samra Z., Kaufman L., Amir J. (2008). Management of nontuberculous mycobacteria-induced cervical lymphadenitis with observation alone. *Pediatric Infectious Disease Journal*.

[47] Griffith D. E., Aksamit T., Brown-Elliott B. A. (2007). An official ATS/IDSA statement: diagnosis, treatment, and prevention of nontuberculous mycobacterial diseases. *American Journal of Respiratory and Critical Care Medicine*.

